# The Development of Novel Organotin Anti-Tumor Drugs: Structure and Activity

**DOI:** 10.1155/MBD.1998.179

**Published:** 1998

**Authors:** Dick de Vos, Rudolph Willem, Marcel Gielen, Kyra E. van Wingerden, Kees Nooter

**Affiliations:** 1 Medical Department Pharmachemie BV PO Box 552 Haarlem NL-003 RN The Netherlands; 2 Free University of Brussels VUB Pleinlaan 2 Department of General and Organic Chemistry Faculty of Engineering Brussels B-1050 Belgium; 3 Free University of Brussels VUB Pleinlaan 2 High Resolution NMR Centre HNMR Brussels B-1050 Belgium; 4 Laboratory for Tumor Biology and Pharmacology Academic Hospital Rotterdam PO Box 2040 Rotterdam NL-3000 CA The Netherlands

## Abstract

An overview of the development of anti-tumor organotin derivatives in selected classes of compounds is presented and discussed. High to very high *in vitro* activity has been found, sometimes equaling that of doxorubicin. Solubility in water is an important issue, dominating the *in vivo* testing of compounds with promising *in vitro* properties. The cytotoxicity of the compounds was increased by the presence of a bulky group, an active substituent or one or more polar substituents. Polar substituents may also improve the water solubility. Although organotin derivatives constitute a separate class of compounds, the comparison with cisplatin is inevitable. Among the observed toxicities, neurotoxicity, known from platinum cytostatics, and gastrointestinal toxicity, typical for many oncology drugs, have been detected. Further research to develop novel, useful organotin anti-tumor compounds should be carried out.


### THE DEVELOPMENT OF NOVEL ORGANOTIN ANTI-TUMOR DRUGS: STRUCTURE AND ACTIVITY

Dick de Vos[1]*, Rudolph Willem[2a,2b], Marcel Gielen[2a],
Kyra E. van Wingerden[3] and Kees Nooter[3]

[1] Medical Department, Pharmachemie BV, PO Box 552, NL-003 RN Haarlem, The Netherlands
[2] Free University of Brussels VUB, Pleinlaan 2, B-1050 Brussels, Belgium
[a] Department of General and Organic Chemistry, Faculty of Engineering
[b] High Resolution NMR Centre HNMR
[3] Laboratory for Tumor Biology and Pharmacology,
Academic Hospital Rotterdam, PO Box 2040, NL-3000 CA Rotterdam, The Netherlands

**Abstract**

An overview of the development of anti-tumor organotin derivatives in selected classes of compounds is presented and discussed. High to very high *in vitro* activity has been found, sometimes equaling that of doxorubicin. Solubility in water is an important issue, dominating the *in vivo* testing of compounds with promising *in vitro* properties. The cytotoxicity of the compounds was increased by the presence of a bulky group, an active substituent or one or more polar substituents. Polar substituents may also improve the water solubility. Although organotin derivatives constitute a separate class of compounds, the comparison with cisplatin is inevitable. Among the observed toxicities, neurotoxicity, known from platinum cytostatics, and gastrointestinal toxicity, typical for many oncology drugs, have been detected. Further research to develop novel, useful organotin anti-tumor compounds should be carried out.

### Introduction

Platinum compounds such as cisplatin [1,2] and carboplatin [3] have found wide application in cancer chemotherapy. Testicular, ovarian and bladder cancer have been treated successfully by combinations containing these drugs. Also small cell lung cancer as well as non small cell lung cancer have been shown responsive to platinum chemotherapy. Also other platinum compounds are under investigation for anti-cancer treatment: e.g. ormaplatin and oxaliplatin [4]. In addition to platinum compounds, derivatives of other metals are being investigated for their anti-tumor properties e.g. titanocene [5].

The disease oriented strategy of the NCI makes use of a disease oriented primary screen. This screen consists of a panel of 60 different human tumor cell lines [6,7]. The NCI screen provides a tool for structure-activity relationships, new members of known mechanistic classes can be found and new mechanistic classes can be discovered. In the present work, the NCI approach was followed and use was made of an *in vitro* primary screen with seven human tumor cell lines, of which five belong to the NCI panel.

The next step is the testing of promising new derivatives in human tumor xenografts in nude mice [8,9]. *In vivo* testing is in general more time consuming than *in vitro* testing. In particular nude mice experiments are rather elaborate due to the nature of the animal and the test and evaluation period. Therefore a murine tumor model was selected. Initially, use was made of the mouse L1210 leukemia [10], later on, the mouse Colon 26 was chosen [11,12]. This model was expected to possess a higher predictive value than the L1210. Subsequently the application of human tumor xenografts in nude mice can be considered for further characterization of the new derivatives.

In the present study the results of the *in vitro* and *in vivo* testing of organotin compounds will be summarized and discussed. As a reference drug, cisplatin will be used. Organotin compounds may yield new leads for the development of anti-tumor drugs, which display another spectrum of antitumor activity, may show non-cross-resistance with platinum drugs and may possess less or different toxicity as compared to platinum compounds.

**Materials and methods**

***Instruments and Procedures***

Instruments and procedures have been described in the papers of the derivatives referred to below.

***Synthesis***

The synthesis and characterization of the compounds have been presented in the references pertaining to the compounds discussed below.

***Antitumor tests***

The following human tumor cell lines have been used in the *in vitro* tests: MCF7 breast cancer, EVSA-T breast cancer, WIDR colon cancer, IGROV ovarian cancer, M19 MEL melanoma, A498 renal cancer and H226 non small cell lung cancer. MCF7 is estrogen receptor (ER)+/progesteron receptor (PgR)+ and EVSA-T is ER-/PgR-. The cell lines WIDR, M19 MEL, A498, IGROV and H226 belong to the anti-cancer screening panel of the National Cancer Institute, USA [6].

Prior to the experiments, a mycoplasma test was carried out on all cell lines and found to be negative. All cell lines were maintained in a continuous logarithmic culture in the standard growth medium RPMI 1640 with Hepes and phenol red. The medium was supplemented with 10% fetal calf serum (FCS), penicillin 100IU/ml and streptomycin 100μg/ml. The cells were mildly trypsinized for passage and for use in the experiments.

RPMI and FCS were obtained from Life Technologies (Paisley, Scotland). Sulforhodamine B (SRB), dimethylsulphoxide (DMSO), ethanol, penicillin and streptomycin were obtained from Sigma (St.Louis, MO, USA), trichloroacetic acid (TCA) and acetic acid from Baker BV (Deventer, NL) and phosphate buffered saline (PBS) from NPBI BV (Emmer-Compascuum, NL).

The test and reference compounds were dissolved to a concentration of 238095 ng/ml in full medium, by 21 fold dilution of an ethanol solution which contained 1 mg of compound/200 μl. Compounds which were found to be insoluble in ethanol were dissolved in DMSO.

The experiments were started on day 0. On day 0, 150 μl of trypsinized tumor cells (1500 - 2000 cells/well) were plated in 96-wells flatbottom microtiter plates (Falcon 3072, BD). The plates were preincubated for 48 hr at 37°C, 8.5% $CO_2$ to allow the cells to adhere. On day 2, a threefold dilution sequence of ten steps was made in full medium, starting with the 238095 ng/ml stock solution. Every dilution was used in quadruplicate by adding 50 μl to a column of four wells. This results in a highest concentration of 59523 ng/ml present in column 12. Column 2 was used for the blank. To column 1, PBS was added to diminish interfering evaporation. On day 7, the incubation was terminated by washing the plate twice with PBS. Subsequently the cells were fixed with 10% trichloroacetic acid in PBS and placed at 4°C for one hour. After five washings with tap water, the cells were stained for at least 15 minutes with 0.4% SRB dissolved in 1% acetic acid. After staining, the cells were washed with 1% acetic acid to remove the unbound stain. The plates were air dried and the bound stain was dissolved in 150 μl 10 mM tris base (tris(hydroxymethyl)aminomethane). The absorbance was read at 540 nm using an automated microplate reader (Labsystems Multiskan MS). Data were used for construction of concentration-response curves and determination of the $ID_{50}$ (dose where 50% of the cells are inhibited) value by use of Deltasoft 3 software. For further details on the test methodology, see refs. [13-15].

In Table I, $ID_{50}$ values of some well known oncology drugs are presented. $ID_{50}$ values may show some variation due to the biological nature of the test. Slight changes in the system during the years of testing may also cause changes in the $ID_{50}$ values. The actual reference values can be found in the papers pertaining to the compounds.

Table I: $ID_{50}$ values (ng/ml) of doxorubicin (DOX), cisplatin (CPT), 5-fluorouracil (5-FU), methotrexate (MTX) and etoposide (ETO)

| | Drug | | | | |
|---|---|---|---|---|---|
| Cell line | DOX | CPT | 5-FU | MTX | ETO |
| MCF7 | 10 | 699 | 750 | 18 | 2594 |
| EVSA-T | 8 | 422 | 475 | 5 | 317 |
| WIDR | 11 | 967 | 225 | <3 | 150 |
| IGROV | 60 | 169 | 297 | 7 | 580 |
| M19 MEL | 16 | 558 | 442 | 23 | 505 |
| A498 | 90 | 2253 | 143 | 37 | 1314 |
| H226 | 199 | 3269 | 340 | 2287 | 3934 |

For the *in vivo* testing, the compounds were dissolved in ethanol or in DMSO, depending on the solubility. Further dilution was either in 2% (w/v) carboxymethylcellulose in saline or in arachidis oil to a concentration of 2 - 10 mg/ml.

The experiments were performed with 10 - 12 week old female Balb/c mice (Harlan/Cpb, Zeist, NL). The animals were housed under standard conditions with water and food *ad libitum*.

MTD (median tolerated dose) studies were performed with groups of 2 mice, which were treated weekly for two weeks by i.p. (intraperitoneal) injection (qdx2). Usually a steep dose - toxicity relation was found. For poorly soluble compounds the toxicity often was unpredictable. This may cause delay in the initiation of anti-tumor experiments.

The murine colon tumor Co 26 (variant Co 26A) was maintained in Balb/c mice by s.c. (subcutaneous) transplantation in both flanks in the thoracic region in small fragments of 1 - 5 $mm^3$. When tumors had reached a volume of 50 - 150 $mm^3$ treatment was started. Tumor size was determined by calliper measurement (length x width x height x 0.5) twice a week. The volume of the tumours was expressed relative to that on the first day of treatment (day 0). Before treatment mice were randomized in groups, one as a control group and the other groups for treatment. Each group consisted of at least 6 mice. Mice were treated by a single i.p. injection. Anti-tumor activity was evaluated by calculation of the T/C (relative tumor size of the treated (T) mice divided by the relative tumor size of the control (C) mice) and the increase of median life span (ILS). Median life span was calculated from the first day of treatment. See ref. [16] for further experimental details. For the murine leukemia L1210, see ref. [10].

*In vitro* tests were carried out in the Laboratory for Tumor Biology and Pharmacology of the Academic Hospital Rotterdam, The Netherlands. *In vivo* tests were carried out by the Department of Medical Oncology of the Free University of Amsterdam, The Netherlands under the supervision of Dr G. J. Peters.

**Results and Discussion**

Many organotin compounds have been synthesized in the past years. From these compounds, a selection will be presented. As a first example of structurally interesting compounds, a series of organotin derivatives of 1,2- and 1,7-dicarba-*closo*-dodecaboranes will be discussed. These novel compounds were prepared from dicarboranyltin dichloride [17,18]. They were tested *in vitro* in the human tumor panel. Their $ID_{50}$ values are summarized in Table II. Compound **1** is o-$C_2B_{10}H_{12}$, compound **2** (m-$C_2B_{10}H_{11}$-9)$_2$Sn$Cl_2$ and compound **6** 2-phenyl-1,2-carborane-1-carboxylic acid.

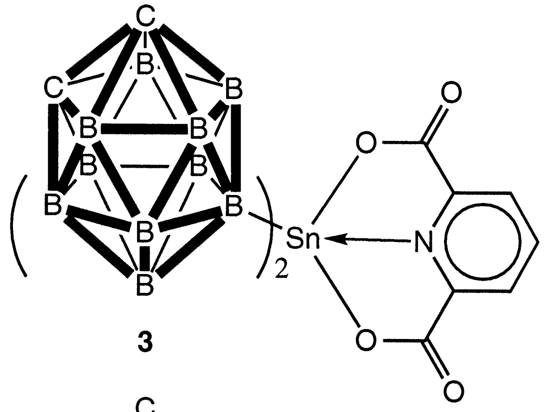

**3**

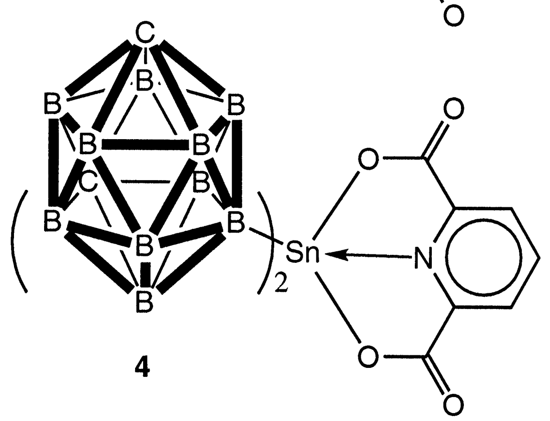

**4**

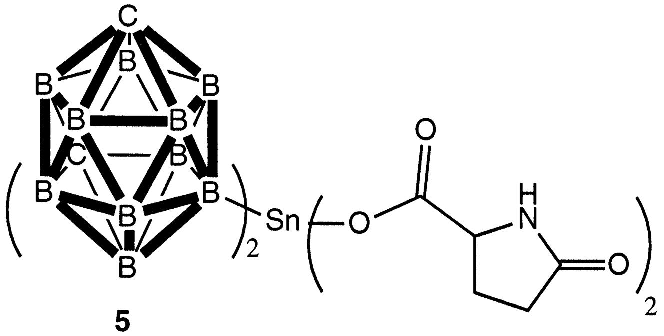

**5**

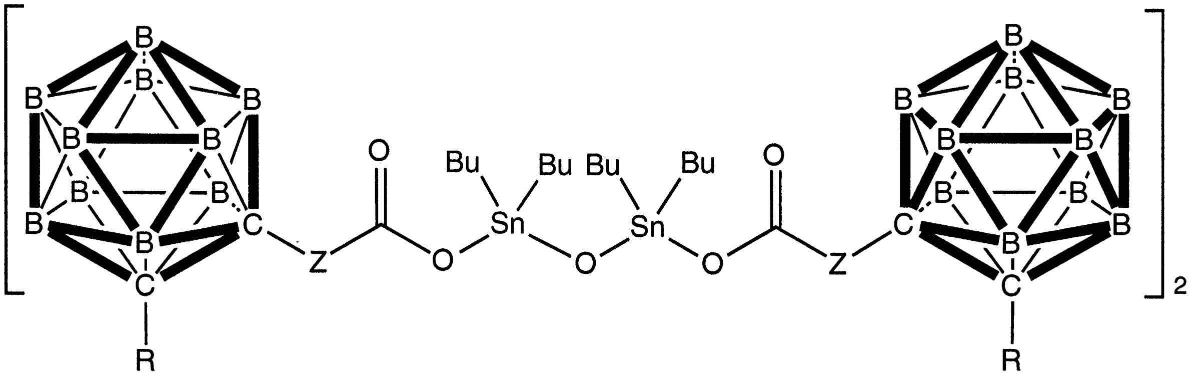

| | | |
|---|---|---|
| **7** | Z = – | R = Ph |
| **8** | Z = $CH_2$ | R = Me |

Table II: $ID_{50}$ values (ng/ml) of some dicarboranyltin compounds

| Compound | Cell line | | | | | | |
|---|---|---|---|---|---|---|---|
| | MCF7 | EVSA-T | WIDR | IGROV | M19 MEL | A498 | H226 |
| **1** | 36817 | 22456 | | | | | |
| **2** | 5 | 31 | | | | | |
| **3** | 14 | 197 | | | | | |
| **4** | 11 | 45 | | | | | |
| **5** | 60 | 48 | 410 | 3 | 30 | 110 | |
| **6** | 56527 | 45168 | 42426 | 58292 | >60000 | 55032 | 11747 |
| **7** | 138 | 164 | 514 | 169 | 220 | 301 | 388 |
| **8** | 74 | 283 | 102 | 172 | 182 | 246 | 140 |

As can be seen from Table II the carboranyltin derivatives show considerable activity compared with the carboranes **1** and **6** and the reference cisplatin. Because of its high activity, compound **4** was tested also *in vivo* in the mouse intraperitoneal (ip) L1210 tumor. Doses of 7, 10, and 14 mg/kg were administered ip. At 10 mg/kg the T/C value was 145 (T/C activity criterium for the L1210 > 125). At the dose of 7 mg/kg there was 1/6 long term survivor.

Another type of active organotin compounds are the triphenyltin derivatives [19]. The *in vitro* activity of two examples is summarized in Table III.

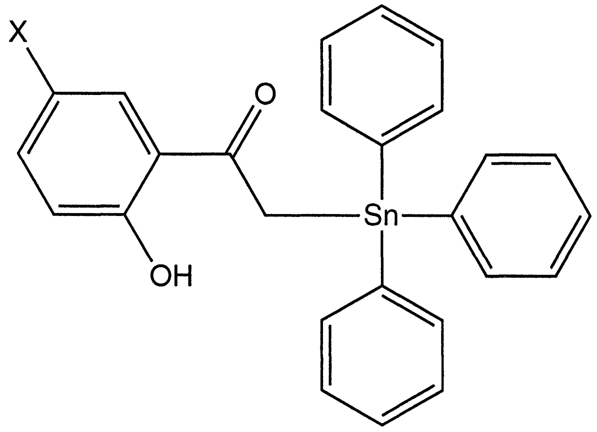

**9** $X = SO_3H$

**10** $X = NH_2$

Table III: $ID_{50}$ values (ng/ml) of two triphenyltin compounds

| Compound | Cell line | | | | | |
|---|---|---|---|---|---|---|
| | MCF7 | EVSA-T | WIDR | IGROV | M19 MEL | A498 |
| **9** | 510 | 400 | 1100 | 290 | 105 | 510 |
| **10** | 200 | 180 | 590 | 490 | 1100 | 700 |

Before compounds **9** and **10** were tested *in vivo* in the murine Co 26, their *in vitro* activity in this tumor was assessed: $ID_{50}$ of compound **9** was 290 and of **10** 97 ng/ml. The $ID_{50}$ of cisplatin was 276 ng/ml. The compounds **9** and **10** were as active *in vitro* as cisplatin or more active. Toxicity testing *in vivo* gave for **9** and **10** MTD values of 5-6 and 8 mg/kg. Although this difference may seem small, compound **9** was highly toxic: paralysis was observed. In the *in vivo* Co 26 compound **9** showed a T/C of 80% and an ILS of 111%, compound **10** gave a T/C of 71% and an ILS of 100%. In this test, the compounds were not active (activity limit for the Co 26 T/C < 42%, ILS > 125%). The T/C of cisplatin, 5.5 mg/kg administered weekly for 4 weeks, was 73% and 39% at the dose of 9 mg/kg.

The test results obtained were encouraging, but there was a practical problem that should be mentioned now already: the solubility. In order to be administered to cancer patients, cytostatics should be soluble in water. Additives can be used to improve the solubility. Also for

*in vivo* testing in animals, the compounds have to possess hydrophilic properties. Compounds **1-10** had to be dispersed in the solvent or dissolved by using an ultra-sonic bath.

Extensive research towards the effect of fluorine substitution in the aryl group of the carboxylate bound to the tin atom has been carried out. A range of fluorine-substituted tin benzoates has been synthesized [20-25] and their *in vitro* antitumor activity assessed. The data of selected compounds have been summarized in Table IV.

Table IV: $ID_{50}$ values (ng/ml) of selected tin fluorine-substituted aromatic carboxylates

| Compound | | Ref. | Cell line | |
|---|---|---|---|---|
| | | | MCF7 | WIDR |
| **11** | $\{[(2\text{-}FC_6H_4COO)(n\text{-}Bu)_2Sn]_2O\}_2$ | 20 | 91 | 330 |
| **12** | $\{[(4\text{-}FC_6H_4COO)(n\text{-}Bu)_2Sn]_2O\}_2$ | 20 | 81 | 360 |
| **13** | $\{[(3\text{-}FC_6H_4COO)(n\text{-}Bu)_2Sn]_2O\}_2$ | 20 | 496 | 3431 |
| **14** | $(3\text{-}FC_6H_4COO)_2Sn(n\text{-}Bu)_2$ | 20 | 39 | 271 |
| **15** | $(2,3\text{-}F_2C_6H_3COO)_2Sn(n\text{-}Bu)_2$ | 22 | 23 | 283 |
| **16** | $\{[(2,3\text{-}F_2C_6H_3COO)(n\text{-}Bu)_2Sn]_2O\}_2$ | 22 | 9 | 120 |
| **17** | $\{[(2,5\text{-}F_2C_6H_3COO)(n\text{-}Bu)_2Sn]_2O\}_2$ | 22 | 7 | 277 |
| **18** | $(3,5\text{-}F_2C_6H_3COO)_2Sn(n\text{-}Bu)_2$ | 22 | 30 | 407 |
| **19** | $\{[(2,6\text{-}F_2C_6H_3COO)(n\text{-}Bu)_2Sn]_2O\}_2$ | 22 | 3 | 174 |
| **20** | $\{[(3,5\text{-}F_2C_6H_3COO)(n\text{-}Bu)_2Sn]_2O\}_2$ | 22 | 11 | 172 |
| **21** | $\{[(2\text{-}FC_6H_4CH{=}CH\text{-}COO)(n\text{-}Bu)_2Sn]_2O\}_2$ | 21 | 28 | 368 |
| **22** | $4\text{-}FC_6H_4COOSnPh_3$ | 19,23 | 15 | 14 |
| **23** | $3\text{-}FC_6H_4COOSnPh_3$ | 23,24 | 10 | 12 |
| **24** | $3,5\text{-}F_2C_6H_3COOSnPh_3$ | 23 | 18 | 17 |
| **25** | $2,3\text{-}F_2C_6H_3COOSnPh_3$ | 23,24 | 31 | 24 |
| **26** | $2,6\text{-}F_2C_6H_3COOSnPh_3$ | 19,25 | 18 | <1 |

The compounds **22** and **26** were also tested *in vivo* in the Co 26 model [19]. Compound **22** gave at a dose of 6 mg/kg a T/C of 67% and an ILS of 111%, compound **26** yielded at a dose of 5 mg/kg a T/C of 87% and an ILS of 111%. Both compounds were judged to be not active in the Co 26 test.

In order to further explore new structural elements and to improve the solubility of the compounds, novel derivatives containing the dihydroxybenzoate [26] and the perfluorobenzoate [27] moieties were prepared. These compounds were tested *in vitro* and found to display promising activity. Some selected results are summarized in Table V.

Table V: $ID_{50}$ values (ng/ml) of dihydroxy- and perfluorobenzoatotin compounds

| Compound | | Cell line | | | | | |
|---|---|---|---|---|---|---|---|
| | | MCF7 | EVSA-T | WIDR | IGROV | M19 MEL | A498 |
| **27** | $[2,4\text{-}(OH)_2C_6H_3COO]_2Sn(n\text{-}Bu)_2$ | 16 | 54 | 120 | 85 | 58 | 130 |
| **28** | $[2,6\text{-}(OH)_2C_6H_3COO]_2Sn(n\text{-}Bu)_2$ | 15 | 58 | 130 | 110 | 65 | 130 |
| **29** | $[2,3\text{-}(OH)_2C_6H_3COO]_2Sn(n\text{-}Bu)_2$ | 7 | 43 | 90 | 51 | 50 | 50 |
| **30** | $[3,5\text{-}(OH)_2C_6H_3COO]_2Sn(n\text{-}Bu)_2$ | 130 | 30 | 500 | 120 | 190 | 280 |
| **31** | $[2,5\text{-}(OH)_2C_6H_3COO]_2Sn(n\text{-}Bu)_2$ | 4 | 48 | 115 | 60 | 65 | 100 |
| **32** | $\{[(C_6F_5COO)(n\text{-}Bu)_2Sn]_2O\}_2$ | 44 | 39 | 214 | 53 | 86 | 76 |
| **33** | $\{[(C_6F_5CH_2COO)(n\text{-}Bu)_2Sn]_2O\}_2$ | 55 | 43 | 275 | 60 | 114 | 105 |
| **34** | $(C_6F_5CH_2COO)_2Sn(n\text{-}Bu)_2$ | 10 | 19 | 145 | 20 | 36 | 50 |
| **35** | $\{[(C_6F_5CH{=}CHCOO)(n\text{-}Bu)_2Sn]_2O\}_2$ | 32 | 37 | 234 | 41 | 66 | 135 |

The introduction of a polar group leads to some improvement in the solubility and definitely to considerable *in vitro* activity. Four of the compounds were tested *in vivo* in the murine Co 26 model [28]. A summary of the results is given in Table VI. Only compound **34** showed modest activity. Toxicity was mainly gastrointestinal.

Table VI: In vivo Co 26 test results of four organotin compounds

| Compound | Dose mg/kg | Schedule | T/C % | ILS % |
|---|---|---|---|---|
| **27** | 6 | qd7x2 | 87 | 100 |
| **31** | 5 | single | 63 | 111 |
| | 5 | qd7x2 | 60 | 97 |
| **33** | 10 | qd7x2 | 120 | 97 |
| **34** | 16 | qd7x2 | 63 | 126 |

Based on earlier work, selected organotin steroidcarboxylates have been synthesized [29]. The new compounds **36-42** have been tested *in vitro* and the results are summarized in Table VII.

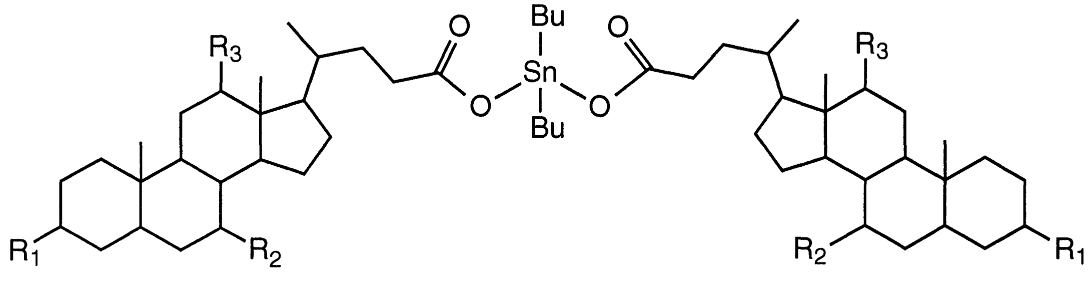

**36** $R_1 = R_3 = OH$; $R_2 = H$ **37** $R_1 = OH$; $R_2 = R_3 = H$ **38** $R_1 = R_2 = R_3$: =O

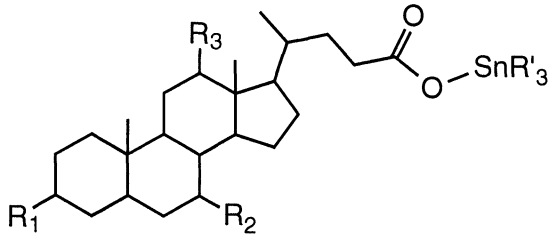

**39** $R_1 = R_3 = OH$; $R_2 = H$; $R' = C_6H_5$

**40** $R_1 = R_3 = R_2$: =O; $R' = C_6H_5$

**41** $R_1 = OH$; $R_2 = R_3 = H$; $R' = C_6H_5$

**42** $R_1 = OH$; $R_2 = R_3 = H$; $R' = n\text{-}C_4H_9$

Table VII: $ID_{50}$ values of selected organotin steroidcarboxylates

| Compound | Cell line | | | | | | |
|---|---|---|---|---|---|---|---|
| | MCF7 | EVSA-T | WIDR | IGROV | M19 MEL | A498 | H226 |
| **36** | 18 | <3 | 36 | 18 | 51 | 42 | 61 |
| **37** | 160 | 60 | 390 | 160 | 120 | 220 | 420 |
| **38** | 409 | 171 | 629 | 150 | 481 | 972 | 1229 |
| **39** | 18 | <3 | 15 | 17 | 32 | 53 | 53 |
| **40** | 11 | <3 | 22 | 16 | 22 | 11 | 50 |
| **41** | 16 | <3 | 19 | 18 | 51 | 65 | 61 |
| **42** | 16 | <3 | 15 | <3 | 51 | 138 | 76 |

The compounds **36-42** displayed appreciable *in vitro* anti-tumor activity. Compounds **36** and **37** were also studied *in vivo* in the murine Co 26 tumor model. The compounds appeared to be so toxic in the tumor bearing mice that a second injection could not be given. The toxicity was higly variable due to the poor solubility of the compounds, which were administered as a dispersion in arachidis oil. The solubility of compound **36** in DMSO was poor, that of compound **37** good. After administration qd7x1 at a dose of 15 mg/kg compound **36** gave a T/C of 42% and an ILS of 8%, compound **37** gave a T/C of 77% and an ILS of 30%.

Compound **36** thus showed activity in the Colon 26 in mice.

The tin steroidcarboxylates appeared to possess considerable *in vitro* anti-tumor activity, but solubility still remained a drawback, which affected their *in vivo* properties. In order to make this type of compounds more soluble, another structure, which contained again a five ring moiety, but now also polar substituents, was designed. This led to the synthesis of organotin terebates [30]. The *in vitro* test results of three compounds have been summarized in Table VIII.

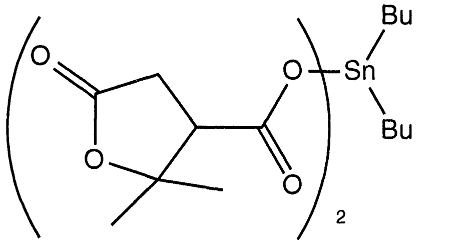

**43**

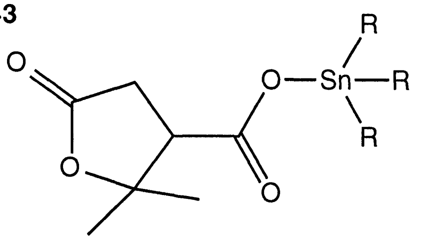

**44** R = n-$C_4H_9$ **45** R = $C_6H_5$

Table VIII: $ID_{50}$ values of some organotin terebates

| Compound | MCF7 | EVSA-T | WIDR | IGROV | M19 MEL | A498 | H226 |
|---|---|---|---|---|---|---|---|
| **43** | 27 | 25 | 134 | 18 | 61 | 61 | 104 |
| **44** | 3 | <3 | 11 | 4 | 11 | 15 | 8 |
| **45** | 17 | <3 | 17 | 19 | 42 | 42 | 39 |

Again the novel organotin compounds were found to have high *in vitro* anti-tumor activity. Compounds **43**-**45** were tested also *in vitro* in the mouse Colon 26 [31]. The solubility of compounds **43** and **44** in DMSO was good, that of **45** poor. The DMSO solution was further diluted with arachidis oil, resulting in a colloidal suspension. The toxicity of the compounds was unpredictable and variable, probably as a result of the limited solubility. There was again considerable toxicity. Only one injection of compound **45** could be given. Two injections of compound **44** resulted in 3/5 toxic deaths in one week. The results of the *in vivo* tests are summarized in Table IX. Some *in vivo* activity was detected.

Table IX: *In vivo* activity of some organotin terebates in the murine Co 26 model

| Compound | Dose mg/kg | Schedule | T/C % | ILS % |
|---|---|---|---|---|
| **43** | 5 | qd7x2 | 91 | 100 |
| **44** | 10 | qd7x2 | 121 | 157 |
| **45** | 15 | qd7x1 | 78 | 100 |

Many organotin compounds have been synthesized during recent years. Test results made clear that considerable *in vitro* activity has been detected in several types of organotin compounds, as has been demonstrated in the selection presented in this paper. Often *in vitro* activity was higher than that of cisplatin and sometimes organotin compounds possessed activity comparable with that of doxorubicin.

The development process led to compounds with definite pharmacological activity: antitumor activity *in vivo*, but also toxicity. Incidentally neurotoxicity, known from cisplatin and oxaliplatin, was detected. The *in vivo* testing was affected by the limited water solubility of the compounds. This is one of the most important factors emerging from the evaluation of the results. The lack of water solubility prevented the use of aqueous solutions in the *in vivo* tests and necessitated the use of arachidis oil for the preparation of a suspension.

The organotin compounds require, as do cisplatin derivatives, a structural moiety containing two substitutable leaving groups forming an angle with the metal, which should lie typically between 90° (cis configuration in cisplatin) and no more than 120°, including the typical tetrahedral geometry (109.5°). The presence of substituents containing a steroid moiety or a carboranyl group enhances the *in vitro* activity. It is not yet clear whether this is the effect of the substituent such as the steroid group or the size of the group. Also the presence of structural units containing a polar substituent contributes to higher activity. A polar substituent may also increase the water solubility of the compounds.

Some compounds such as the organotin terebates merit more detailed investigation. Further chemical and pharmacological studies are necessary to unravel a structure-activity relationship from which novel organotin anti-tumor drugs for use in patients can be developed.

**References**

1. P.J. Loehrer, L.H. Einhorn, *Ann. Intern. Med.*, 1984, **100**, 704.
2. R.L. Comis, *Sem. Oncol.*, 1994, **21**, suppl. 12, 109.
3. J.C. Ruckdeschel, *Sem. Oncol.*, 1994, **21**, suppl. 12, 114.
4. R.B. Weiss, M.C. Christian, *Drugs,* 1993, **46**, 360.
5. C.M. Kurbacher, P. Mallmann, J.A. Kurbacher, G. Sass, P.E. Andreotti, A. Rahmun, H. Hubner, D. Krebs, *Anticancer Res.,* 1994, **14**, 1961.
6. M.R. Boyd, Cancer: *Principles and practice of oncology updates*, V.T. de Vita, Jr, S. Hellman, S.A. Rosenberg, eds, 1989, **3**, 10, 1.
7. M.R. Boyd, K.D. Paull, *Drug Dev. Res.*, 1995, **34**, 91.
8. B. Winograd, E. Boven, M.W. Lobbezoo, H.M. Pinedo, *In vivo,* 1987, **1**, 1.
9. E. Boven, B. Winograd, D.P. Berger, M.P. Dumont, B.J.M. Braakhuis, O. Fodstad, S. Langdon, H.H. Fiebig, *Cancer Res.*, 1992, **52**, 5940.
10. P. Lelieveld, L.M. van Putten, *Cancer Treat Rep.*, 1976, **60**, 373.
11. T.H. Corbett, D.P. Griswold, B.J. Roberts, J.C. Peckham, F.M. Schabel, *Cancer,* 1977, **40**, 2660.
12. P.J. van Kranenburg-Voogd, H.J. Keizer, L.M. van Putten, *Eur. J. Cancer*, 1978, **14**, suppl., 153.
13. P. Skehan, R. Storeng, D. Scudiero, A. Monks, J. McMahon, D. Vistica, J.T. Warren, H. Bokesch, S. Kenney, M.R. Boyd, *J. Natl. Cancer Inst.*, 1990, **13**, 1107.
14. L.V. Rubinstein, R.H. Shoemaker, K.D. Paull, R.M. Simon, S. Tosini, P. Skehan, D.A. Scudiero, A. Monks, M.R. Boyd, *J. Natl. Cancer Inst.*, 1990, **13**, 1113.
15. Y.P. Keepers, P.E. Pizao, G.J. Peters, J. van Ark-Otte, B. Winograd, H.M. Pinedo, *Eur. J. Cancer,* 1991, **27**, 897.
16. J.A.M. van Laar, Y.M. Rustum, C.L. van der Wilt, K. Smid, C.M. Kuiper, H.M. Pinedo, G.J. Peters, *Cancer Chemother. Pharmacol.*, 1996, **39**, 79.
17. M. Gielen, F. Kayser, O.B. Zhidkova, V.Ts. Kampel, V.I. Bregadze, D. de Vos, M. Biesemans, B. Mahieu, R. Willem, *Metal Based Drugs,* 1995, **2**, 37.
18. E.R.T. Tiekink, M. Gielen, A. Bouhdid, R. Willem, V.I. Bregadze, L.V. Ermanson, S.A. Glazon, *Metal Based Drugs, 1997, **4**, 75.*
19. M. Gielen, R. Willem, A. Bouhdid, D. de Vos, C.M. Kuiper, G. Veerman, G.J. Peters, *In Vivo*, 1995, **9**, 59.
20. M. Gielen, A. El Khloufi, M. Biesemans, R. Willem, *Appl. Organomet. Chem.*, 1993, **7**, 119.
21. M. Gielen, A. El Khloufi, M. Biesemans, F. Kayser, R. Willem, *Appl. Organomet. Chem.*, 1993, **7**, 201.

22. M. Gielen, M. Biesemans, A. El Khloufi, J. Meunier-Piret, F. Kayser, R. Willem, *J. Fluor. Chem.*, 1993, **64**, 279.
23. M. Gielen, *Coord. Chem. Rev.*, 1996, **151**, 41.
24. M. Gielen, A. El Khloufi, M. Biesemans, A. Bouhdid, D. de Vos, B. Mahieu, R. Willem, *Metal Based Drugs,* 1994, **1**, 305.
25. M. Gielen, A. El Khloufi, D. de Vos, H.J. Kolker, J.H.M. Schellens, R. Willem, *Bull. Soc. Chim. Belg.*, 1993, **102**, 761.
26. M. Gielen, A. Bouhdid, F. Kayser, M. Biesemans, D. de Vos, B. Mahieu, R. Willem, *Appl. Organomet. Chem.*, 1995, **9**, 251.
27. M. Gielen, E.R.T. Tiekink, A. Bouhdid, D. de Vos, M. Biesemans, I. Verbruggen, R. Willem, *Appl. Organomet. Chem.*, 1995, **9**, 639.
28. M. Gielen, R.Willem, A. Bouhdid, D. de Vos, C.M. Kuiper, G. Veerman, G.J. Peters, *Oncol. Rep.*, 1996, **3**, 583.
29. R. Willem, H. Dalil, P. Broekaert, M. Biesemans, L. Ghys, K. Nooter, D. de Vos, F. Ribot, M. Gielen, *Main Group Met. Chem.*, 1997, **20**, 535.
30. M. Gielen, H. Ma, A. Bouhdid, H. Dalil, M. Biesemans, R. Willem, *Metal Based Drugs,* 1997, **4**, 193.
31. M. Gielen, R. Willem, H. Dalil, D. de Vos, C.M. Kuiper, G.J. Peters, *Metal Based Drugs,* 1998, **5**, 83.

**Received: June 18, 1998 - Accepted: July 14, 1998**

